# Electronic measurement of frequency and duration of close nurse–patient contacts during patient care in intensive and intermediate care units: a cross-sectional observational study

**DOI:** 10.1016/j.infpip.2026.100534

**Published:** 2026-03-24

**Authors:** M.M. Neuwirth, M. Neuwirth, C. Karagiannidis, F. Mattner, R. Otchwemah

**Affiliations:** a Hygiene and Environmental Medicine, Faculty of Health/Department of Human Medicine, University Witten/Herdecke, Cologne, Germany; b Institute for Hygiene, Cologne Merheim Medical Centre, University Hospital Witten/Herdecke, Cologne, Germany; c Central Department of Hygiene and Environmental Medicine, University Hospital Essen, University of Duisburg-Essen, Essen, Germany; d Institute for Electromobility, Bochum University of Applied Sciences, Bochum, Germany; e ARDS and ECMO Centre Cologne Merheim, University Witten/Herdecke, Cologne, Germany; f Pneumology, Faculty of Health/Department of Human Medicine, University Witten/Herdecke, Witten, Germany

**Keywords:** Patient–nurse interaction, Healthcare worker safety, Infection prevention, Nursing staff exposure, Bluetooth low energy transmitter, Close contact

## Abstract

**Background:**

Healthcare workers face elevated infection risks by droplet or airborne transmissible pathogens during close patient care, but objective data on actual exposure patterns are scarce.

**Methods:**

We measured nurse–patient contacts (less than 1.5 metres from the patient’s head) using Bluetooth Low Energy transmitters on one respiratory intensive care unit (ICU) (December 2020–February 2021) and one respiratory intermediate care unit (IMU) (August 2021) at a German university hospital. Contact frequency, average duration, and cumulative exposure time per 8-h shift were analysed by ward and work shift.

**Results:**

ICU nurses had on average 80.3 contacts per shift (mean duration = 46.8 s); IMU nurses had 27.9 contacts (43.6 s). Cumulative time <1.5 metres was 31.8 min in the ICU vs 9.6 min in the IMU. Exposure peaked during early shifts in both units. Differences between ICU and IMU were statistically significant (*P* < 0.05; Cohen’s d > 1.2).

**Conclusion:**

This study provides the first objective measurements of nurse–patient exposure in ICU and IMU care. ICU nurses experienced substantially higher frequency and duration of close contacts.

## Introduction

Healthcare workers are exposed to significant occupational risks from the transmission of respiratory viruses that may lead to respiratory infections. This challenge did not begin with the Coronavirus disease 2019 (COVID-19) pandemic but was evident during previous outbreaks. During the SARS epidemic of 2003, healthcare workers accounted for 20% of all probable cases worldwide [1]. The situation was similar during the H1N1 influenza pandemic in 2009, when healthcare workers had more than twice the risk of infection than control groups [2]. These examples illustrate the high risk for healthcare workers, especially nursing staff, of becoming infected with respiratory viruses. In addition, there are seasonal influenza, respiratory syncytial virus (RSV), and severe acute respiratory syndrome coronavirus 2 (SARS-CoV-2), which, unlike one-off outbreaks, pose an annually recurring risk of nosocomial transmission to healthcare workers [3].

Respiratory viruses are mainly transmitted via droplets (>5–10 μm) and aerosols (<5 μm), which lead to high infectivity. However, the probability of infection via this transmission route generally decreases with increasing distance from the source of infection [4]. The historically and empirically established guideline recommending a distance of 1–2 m for infection prevention and control is mostly based on measurements and studies that show that the concentration of infectious droplets and aerosols decreases significantly with increasing distance, thereby significantly reducing the risk of exposure to infectious particles [[5], [6], [7], [8]]. In experimental setups with simulated coughing events, the particle concentration decreased by about 80% at 1.5 m compared with 0.5 m [4]. Aerosol models also showed that virus-laden particles disperse rapidly even at short distances, regardless of their size [9]. This principle applies regardless of the pathogen, be it tuberculosis, influenza, or coronaviruses, and is therefore often a basic assumption for personal protective measures.

The COVID-19 pandemic has reinforced these historical patterns and made them more visible. Most recently, SARS-CoV-2 infections acquired in hospitals played a significant role during the COVID-19 pandemic. This included infections in both patients and medical staff. At the onset of the pandemic in 2020, infections among medical staff acquired during work were prevalent. In Wuhan, 29% of hospitalized COVID-19 cases were medical personnel [10]. At the onset of the 2020 outbreak in Lombardy, up to 20% of physicians and nurses were infected [11]. In March 2020, Germany experienced infected medical staff at one of the largest university perinatal centres in Bavaria, with 36 staff members affected, including physicians, nurses, and midwives [12]. SARS-CoV-2 is mainly transmitted via droplets and aerosols, resulting in high infectivity and documented transmission in hospitals [13,14].

As direct patient care involves frequent and close contact with patients, nursing staff members are particularly susceptible to respiratory infections transmitted by droplets [15,16].

Until now, there has been a lack of objective measurement data on the actual exposure of nursing staff [17]. To address this gap, this study aimed to determine the frequency and duration of close patient contacts (less than 1.5 m) among nurses in an intensive care unit (ICU) (mainly SARS-CoV-2-positive patients) and an intermediate care unit (IMU) (mainly SARS-CoV-2-negative patients) during different work shifts (early, late, and night shifts).

## Methods

This study was designed as a cross-sectional observational study using continuous Bluetooth Low Energy (BLE)–based proximity monitoring, conducted during two separate measurement periods.

All nurses actively employed on the respective ward during the measurement period were eligible for participation. Inclusion required provision of direct patient care and willingness to wear the BLE wearable throughout the full shift. No exclusion criteria based on professional role, seniority, or shift type were applied. Regarding patients, all beds equipped with BLE transmitters and occupied during the measurement period were included; no patient-level clinical selection criteria were applied as only anonymized proximity data were recorded.

During the first measurement period (14^th^ December 2020 to 28^th^ February 2021), 12 beds in the respiratory ICU of a German university hospital were equipped with BLE transmitters (CLINARIS GmbH, Germany), where the majority of patients were diagnosed with COVID-19 infection. The second measurement period (1^st^ August to 31^st^ August 2021) took place in a respiratory IMU of the same hospital, where 17 beds were equipped with BLE transmitters in the same manner.

**Figure 1:**
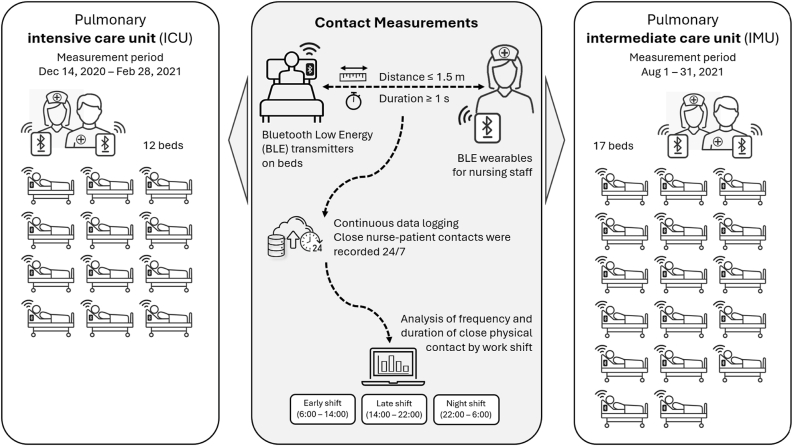
Schematic overview of the BLE-based contact measurement setup. Bluetooth Low Energy (BLE) transmitters are placed at the head end of each bed, and nurses wear BLE receivers to record close contacts (<1.5 m). Data are logged continuously (24/7) and analysed by work shift (early, late, and night shifts).

In both wards, nursing staff members were provided with additional BLE wearables (CLINARIS GmbH, Germany) to record the frequency, duration, and distance of patient contacts. Distance was defined as the nurse’s position relative to the head end of the patient’s bed. BLE devices enabled wireless communication of small data packets over short distances with minimal energy consumption (see Figure 1 for a schematic overview of the measurement setup).

Participation in the measurements was voluntary, and not all nurses wore BLE wearables. The wearables were attached uniformly to the breast pocket of the work clothing and worn throughout the entire shift. Nurses who chose not to participate were not recorded.

The primary outcomes were (1) contact frequency (mean number of close contacts per 8-h shift per nurse), (2) mean contact duration (mean duration in seconds per individual contact event), and (3) total contact time per shift (sum of durations of all contact events in minutes). All outcomes were analysed stratified by ward type (ICU vs IMU) and work shift (early, late, and night shifts). No secondary outcomes were prespecified.

Only measurement data that fulfilled the following criteria were analysed: A contact was defined as a threshold value being exceeded at least twice in succession at a distance of no more than 1.5 m from the patient’s head for a duration of at least 1 second. Events shorter than 1 s were discarded. Gaps longer than 1 second terminated the contact event.

The distance was determined using the Received Signal Strength Indication (RSSI) value. A minimum RSSI threshold of −55 decibels milliwatts (dBm) was defined, which corresponds to a distance of approximately 1.5 m from the bed-mounted BLE transmitter. This threshold was validated by electronically measuring the signal strength at various distances between the nurses and the head, foot, and side of the bed. Before the data collection phase began, all BLE transmitters and BLE wearables were calibrated together to match transmitter power and receiver sensitivity and ensure consistent detection of interactions.

Data collection was continuous (24 h a day, 7 days a week). For analysis, the data were divided into three intervals: early shift (06:00–14:00), late shift (14:00–22:00), and night shift (22:00–06:00).

The number of contacts per shift per nurse was calculated as the mean number of individual contacts per shift. The average contact duration per shift per patient was calculated as the mean duration of individual contacts for each nurse during a shift. During longer nursing activities (e.g. suctioning, washing, and caring for the central venous catheter), it could happen that the presence within 1.5 m was logged as several individual contacts. This occurred when the caregiver left the relevant measurement range of 1.5 m for longer than 1 second and then re-entered it, resulting in separate events being counted. Therefore, the average contact duration represents the mean value of the technically defined events and not necessarily the total uninterrupted duration of a nursing activity.

Total contact time under 1.5 m per shift was calculated as the sum of the durations of all individual contacts within the defined distance threshold.

All participating nurses provided patient care wearing full personal protective equipment (PPE) throughout the study period due to the ongoing COVID-19 pandemic.

Potential sources of bias were considered during study design and analysis. Selection bias due to voluntary participation was addressed by including all consenting nurses without restriction. A potential Hawthorne effect cannot be excluded but is unlikely to have substantially distorted the results: the continuous measurement periods lasted up to 11 weeks, and behavioural adaptation to wearable monitoring has been shown to diminish markedly after the first few days of use. Technical measurement bias was mitigated through standardized prestudy calibration of all BLE devices. Confounding by varying staff-to-patient ratios across shifts was not adjusted for statistically as this study was descriptive in nature.

For comparisons of mean values between the ICU and IMU, *t*-tests with pooled variance were used for comparisons where equal variances were assumed. Welch *t*-tests were performed for comparisons with unequal variances (Levene *P* < 0.05). The degrees of freedom for Welch tests were Satterthwaite corrected. Effect sizes are reported as Cohen’s d. Negative d values indicate fewer contacts in the IMU than in the ICU.

No formal a-priori power calculation was performed as this was a descriptive observational study without a prespecified primary hypothesis test. The sample size was determined by operational constraints: all nurses employed on the two wards who consented to participate were enrolled. The resulting sample (ICU: 17 nurses, 56 patients; IMU: 10 nurses, 43 patients) is appropriate for the exploratory aims of this study but should be considered a limitation when generalizing findings to other settings.

## Results

Of the nursing staff employed on both wards during the respective measurement periods, all who provided informed consent were enrolled. A total of 27 nurses wore BLE wearables (ICU: n=17; IMU: n=10). No participants withdrew during the study. Individual shift records without full-shift BLE data (e.g. due to forgotten wearable or device malfunction) were excluded from shift-level analyses; the exact number of such excluded records was not separately tracked.

During the study period, 56 patients were cared for by 17 nurses wearing BLE wearables in the ICU and 43 patients by 10 nurses in the IMU in total. The key quantitative findings on nurse–patient close contacts (<1.5 m) are summarized in Table I.

**Table I:** Results of the electronic measurement of close nurse–patient contacts (<1.5 m) in the ICU and IMU.

Shift	Ward type (*N* = Number of nursing staff)	Mean Number of contacts (±SD) (per shift/nurse to patients)	*t* (*df*)	*P*	Cohen *d*	Average duration of a single contact in seconds (±SD) (per shift/nurse to patients)	*t* (*df*)	*P*	Cohen’s *d*	Total contact time in minutes (±SD) (per shift/nurse to patients)	*t* (*df*)	*P*	Cohen’s *d*
**Early shift** (6:00–14:00)	Intensive care unit (*n* = 10)	91.13 (±52.90)	−2.81 (10.15)	.018∗	−1.27	48.17 (±11.17) s	0.29 (16)	.777	0.14	38.08 (±27.77) min	−3.13 (10.96)	.010 ∗∗	−1.21
	Intermediate care unit (*n* = 7)	38.13 (±17.52)				49.52 (±6.61) s				11.27 (±4.89) min			
**Late shift** (14:00–22:00)	Intensive care unit (*n* = 10)	78.63 (±35.04)	−2.90 (14)	.012∗	−1.49	43.29 (±9.42) s	−0.75 (13)	.465	−0.40	32.41 (±17.84) min	−2.10 (13)	.056	−1.11
	Intermediate care unit (*n* = 6)	32.75 (±20.70)				39.94 (±6.56) s				14.63 (±12.71) min			
**Night shift** (22:00–6:00)	Intensive care unit (*n* = 13)	52.54 (±29.73)	−3.74 (9.36)	.004∗∗	−1.54	45.11 (±14.88) s	−0.43 (15)	.674	−0.21	20.25 (±13.34) min	−3.49 (9.50)	.006 ∗∗	−1.43
	Intermediate care unit (*n* = 7)	17.02 (±3.54)				42.46 (±7.91) s				5.32 (±1.87) min			
**Total**	Intensive care unit (*n* = 11)	80.31 (±25.44)	−6.36 (12.82)	< .001∗∗∗	−2.53	46.82 (±10.85) s	−0.92 (12.16)	.375	−0.36	31.84 (±15.97) min	−4.38 (12.03)	.001 ∗∗∗	−1.72
	Intermediate care unit (*n* = 7)	27.86 (±7.98)				43.63 (±2.93) s				9.64 (±4.17) min			

*Note.* Contact defined as distance <1.5 m from patient’s head.

Statistical tests: Welch’s *t*-test was used where variances were unequal (Levene *P* < .05); the pooled-variance *t*-test was used otherwise.

Degrees of freedom (*df*) adjusted with Satterthwaite method for Welch’s *t*-tests.

Cohen’s *d* effect size interpretation: 0–0.19 = trivial, 0.20–0.49 = small, 0.50–0.79 = medium, ≥0.80 = large; negative d indicates lower means in the IMU than in the ICU.

*∗P*< .05; ∗∗*P*< .01; ∗∗∗*P*< .001.

*N* refers to the number of nurses with complete shift-level BLE recordings for the respective shift type. As not all nurses worked every shift type during the measurement period, shift-specific *N* values are smaller than the total number of enrolled nurses (ICU = 17; IMU = 10). The total row reflects the overall mean across all recorded shifts per nurse.

The table presents the mean number of contacts per shift, the average duration of individual contacts, and the total contact time per shift. Data are stratified by ward type and work shift (early, late, and night shifts).

ICU, intensive care unit; IMU, intermediate care unit; SD, standard deviation; BLE, Bluetooth Low Energy.

On average, a nurse had 73.6 relevant close patient contacts per 8-hour shift across both units, with a mean contact duration of 45.7 s per contact. Comparing the two wards on an individual basis, nurses in the ICU had significantly more close contacts than those in the IMU (mean = 80.3 vs 27.9 contacts per shift, *P* < 0.05). It was observed that the duration of contacts in the ICU (46.8 s) was slightly longer than in the IMU (43.6 s). Accordingly, the total time spent within 1.5 m of patients’ heads was significantly longer in the ICU, averaging 31.8 min per shift, than 9.6 min in the IMU.

The analysis of work shifts revealed that the majority of contacts, and also the longest contact durations, occurred during the early shift in both wards. ICU nurses exhibited an average of 91.1 contacts per early shift, with each contact lasting approximately 48.2 s. In comparison, IMU nurses had 38.1 contacts, with an average duration of 49.5 s. Contact frequency and duration of contact decreased progressively during the late and night shifts in both units. The total time spent in close contact per shift showed a similar trend: ICU nurses spent an average of 38.1 min in close contact during the early shift, while IMU nurses spent 11.3 min in close proximity to patients (see Table I).

Statistical comparisons between the ICU and IMU demonstrated significant differences in the number of close contacts and total contact times across all shifts (*P* < 0.05), with large effect sizes (Cohen’s d > 1.2) indicating considerably higher exposure in the ICU (see Table I).

## Discussion

This study provides novel, objective data on the frequency and duration of relevant close contacts (<1.5 m) between nurses and patients in both respiratory ICU and IMU settings during the COVID-19 pandemic. These results contribute to a better assessment of occupational exposure risk among nursing staff.

ICU nurses had significantly more close patient contacts per shift (80.3) than IMU nurses (27.9), with longer cumulative contact times (31.8 vs 9.6 min per shift). As in other studies, healthcare staff members with many patient contacts are most at risk [[18], [19], [20]].

The highest contact frequencies and durations were observed during the early shifts in both units, which is consistent with the findings of Vanhems *et al.* (2013) [21], who reported a peak in nursing activities in the morning.

The higher exposure in the form of more frequent contact and longer total contact time in the ICU indicates that caring for critically ill patients, especially COVID-19-infected patients, is associated with more intensive care [[22], [23], [24]]. The higher frequency and cumulative duration of close contacts observed in the ICU are consistent with the therapeutic requirements of critically ill patients, who often need invasive ventilation, continuous monitoring, vasoactive therapy, and repeated procedures such as suctioning or managing central venous and arterial lines. In contrast, IMU patients are usually less unstable and more mobile. They often self-ventilate with non-invasive or low-flow oxygen support, which typically requires fewer and shorter bedside procedures. This results in fewer recorded close contacts per shift.

This distinction underscores the heterogeneous workloads per patient and occupational risks between the different wards [25]. In addition, differences in the patient population and the ratio of nursing staff to patients, as well as in the workflows between the ICU and the IMU, could have an impact on contact patterns.

ICU nurses spent on average about 32 min per shift within 1.5 m of a patient’s head, which is about 6.5% of a standard 8-hour shift. The duration of close contact of 32 min is comparable with the result of a survey of healthcare workers who stated that they had been in contact with presumably COVID-19-infected patients for more than 30 min [18].

This sustained proximity exposes nursing staff to a significant risk of droplet and aerosol transmission of pathogens like SARS-CoV-2 [8,14].

Despite the shorter exposure time in the IMU, the frequency of close contact between nursing staff and patients remains high, reinforcing the ongoing need to adhere to infection control standards in all areas and wards with patient care.

A major limitation of this study is its single-centre design restricted to only two respiratory wards within one hospital, which limits generalizability. A further limitation is that ICU and IMU measurements were conducted during different calendar periods, which may have influenced workflows, staffing, and infection control practices independently of the ward type. Furthermore, while our measurements quantify exposure time during close contact, they do not directly capture the probability of pathogen transmission. For instance, many ICU patients were invasively ventilated using closed, filtered circuits, which could reduce the emission of infectious respiratory particles, despite nurses having higher exposure times at the bedside. Conversely, self-ventilating ICU patients with symptomatic respiratory infections (e.g. coughing or sneezing) could pose a higher transmission risk per unit of exposure time in individual situations despite lower cumulative close contact time than ICU patients. When interpreting our findings for infection prevention strategies, this distinction between exposure intensity and transmission probability should be considered.

Additional limitations include the small sample size of nurses and the technical inaccuracy of RSSI-based distance estimation.

Since the technical recording of contacts was based on successive signal events, longer activities in patient care could be represented as several shorter contacts if the nurse briefly left the 1.5-m distance threshold and then re-entered it. This means that the average contact duration in the data set appears relatively short, even though activities such as washing, suctioning, or caring for vascular accesses often take several minutes in practice.

This methodological definition of the technical recording of contacts allows for standardized evaluation but should also be seen as a limitation in interpretation.

The number of staff members per shift also varied over time, meaning that not every measurement period included the same number of nurses per shift. As enrolment was voluntary, the recorded sample captures only nurses who consented to wear the device. It cannot be determined whether non-participating nurses had systematically different contact patterns, for example, due to different task profiles or shift preferences.

The results show the number of relevant contacts with infection risk of respiratory viruses that nurses have when caring for intensive and intermediate care patients. This provides indications for assessing the risk of infection, and based on this, guidance for wearing PPE in the daily work of ICUs and IMUs. Wearable technical devices may support healthcare workers in risk assessment and analysis of work processes. Future studies with larger samples and multi-centre designs are needed to validate these results.

## CRediT authorship contribution statement

**M.M. Neuwirth:** Writing – original draft, Visualization, Project administration, Investigation, Formal analysis, Data curation. **M. Neuwirth:** Formal analysis, Data curation. **C. Karagiannidis:** Writing – review & editing, Supervision, Conceptualization. **F. Mattner:** Writing – review & editing, Supervision, Methodology, Conceptualization. **R. Otchwemah:** Writing – review & editing, Supervision, Methodology, Conceptualization.

## Ethics statement

The present study involved anonymized, observational measurement of the frequency and duration of close contacts between nursing staff and patients in intensive and intermediate care units. Only spatial distance data were recorded; no personally identifiable information of patients or staff was collected.

According to the Rules of Procedure of the Ethics Committee of Witten/Herdecke University (version dated January 8, 2015), ethical approval is mandatory for clinical trials and biomedical research involving direct human participation or personal patient data. The primary purpose of the Ethics Committee is to protect patients involved in clinical trials and interventional studies. As this study did not involve patient data or interventions and solely collected anonymized observational data, it does not meet the criteria requiring formal ethical review.

The Ethics Committee of Witten/Herdecke University (Ethik-Kommission der Universität Witten/Herdecke e.V.) was consulted and confirmed that no formal application or ethical approval was necessary for this observational study in accordance with their Rules of Procedure (§ 2 Antrag, Abs. 1). Therefore, ethical approval was waived on the basis that no formal application was submitted, as allowed by the institutional regulations.

All participating nursing staff members were fully informed about the purpose and procedures of the study. Informed consent to participate was obtained from all participants, with the right to withdraw at any time without providing reasons. Approval from the local works council was additionally obtained to ensure compliance with institutional and labour policies.

This study complies with the ethical principles of the Declaration of Helsinki, relevant national recommendations such as those of the German Society for Nursing Science, and the European General Data Protection Regulation.

## Consent for publication

No images or personal or clinical details of participants or patients that could compromise anonymity are displayed. Therefore, no declaration of consent from participants or patients is required for publication.

## Availability of data and materials

As part of our measurement, we collected anonymized measurement data on the distance between nursing staff and patients. However, for reasons of data protection and ethics, we do not intend to publish the raw data. Despite anonymization, there is a residual risk that individual data points could be traced back to specific individuals by comparing them with work-related information, such as duty rosters. The works council was explicitly assured that no such comparison would be made, thus ruling out identification of individuals.

Furthermore, without specific processing, third parties cannot understand or interpret the raw data, which could lead to misinterpretation and misuse. For these reasons, the raw data will not be published.

## Funding sources

No funding was received for this study.

## Conflict of interest statement

The authors declare that they have no competing interests.
